# *MGMT*, *GATA6*, *CD81*, *DR4*, and *CASP8* gene promoter methylation in glioblastoma

**DOI:** 10.1186/1471-2407-12-218

**Published:** 2012-06-06

**Authors:** Daina Skiriute, Paulina Vaitkiene, Viktoras Saferis, Virginija Asmoniene, Kestutis Skauminas, Vytenis Pranas Deltuva, Arimantas Tamasauskas

**Affiliations:** 1 Laboratory of Neurooncology and Genetics, Neuroscience Institute, Medical Academy, Lithuanian University of Health Sciences, Eiveniu str. 4, Kaunas, LT, 50009, Lithuania; 2 Department of Physics, Mathematics, and Biophysics, Medical Academy, Lithuanian University of Health Sciences, Eiveniu str. 4, Kaunas, LT, 50009, Lithuania

**Keywords:** *MGMT*, *CD81*, *GATA6*, *DR4*, *CASP8*, DNA methylation, MSP, Glioblastoma, Survival

## Abstract

**Background:**

Methylation of promoter region is the major mechanism affecting gene expression in tumors. Recent methylome studies of brain tumors revealed a list of new epigenetically modified genes. Our aim was to study promoter methylation of newly identified epigenetically silenced genes together with already known epigenetic markers and evaluate its separate and concomitant role in glioblastoma genesis and patient outcome.

**Methods:**

The methylation status of *MGMT*, *CD81*, *GATA6*, *DR4*, and *CASP8* in 76 patients with primary glioblastomas was investigated. Methylation-specific PCR reaction was performed using bisulfite treated DNA. Evaluating glioblastoma patient survival time after operation, patient data and gene methylation effect on survival was estimated using survival analysis.

**Results:**

The overwhelming majority (97.3%) of tumors were methylated in at least one of five genes tested. In glioblastoma specimens gene methylation was observed as follows: *MGMT* in 51.3%, *GATA6* in 68.4%, *CD81* in 46.1%, *DR4* in 41.3% and *CASP8* in 56.8% of tumors. Methylation of *MGMT* was associated with younger patient age (p < 0.05), while *CASP8* with older (p < 0.01). *MGMT* methylation was significantly more frequent event in patient group who survived longer than 36 months after operation (p < 0.05), while methylation of *CASP8* was more frequent in patients who survived shorter than 36 months (p < 0.05). Cox regression analysis showed patient age, treatment, *MGMT*, *GATA6* and *CASP8* as independent predictors for glioblastoma patient outcome (p < 0.05). *MGMT* and *GATA6* were independent predictors for patient survival in younger patients’ group, while there were no significant associations observed in older patients’ group when adjusted for therapy.

**Conclusions:**

High methylation frequency of tested genes shows heterogeneity of glioblastoma epigenome and the importance of *MGMT*, *GATA6* and *CASP8* genes methylation in glioblastoma patient outcome.

## Background

Glioblastoma is the most common and aggressive astrocytic tumor of the central nervous system in adults. It is characterized by various genetic alterations, affecting genes that control cell growth, apoptosis, angiogenesis, and invasion. Epigenetic alterations also affect the expression of cancer genes alone or in combination with genetic mechanisms. Cytosine methylation of CpG dinucleotides in gene promoters is a common cause of DNA silencing and transcriptional repression that can modulate clinical features of globlastoma. The best known is O^6^-methylguanine-DNA methyltransferase (*MGMT*) promoter methylation determining tumors response to DNA alkylating agents and being an independent prognostic factor for patient survival [1]. However, several widely described genes can be seen as only a partial picture of the methylation changes, there may be many more genes that need to be clarified. Recent methylome studies of brain tumors have disclosed a list of new epigenetically modified genes associated with gliomagenesis and different glioma clusters [2-4]. The latter studies illustrate glioblastoma profile being constructed via methylation of a multiple set of genes, forming networks attributed to different pathogenesis pathways. It has been suggested that in glioblastoma, highly methylated genes, such as *CD81* (CD81 antigen), *DR4* (death receptor 4, TRAIL receptor 1) and *GATA6* (GATA binding protein 6), participating in cell adhesion, apoptosis, and proliferation [3] could be important in gliomagenesis. Newly obtained influence of *GATA6* hypermethylation on glioblastoma patient survival in a small group of tumors was shown as a promising marker in prognostic purposes [3]. To further study the importance of promoter methylation of newly identified epigenetically silenced genes (*GATA6CD81*) and revealing its relationship with already known epigenetic markers (*MGMTDR4CASP8* (caspase-8)) could help to better understand glioblastoma biology. In the first part of the study we have reported promoter methylation of *MGMTGATA6CD81DR4*, and *CASP8* genes. In the second part we have analyzed gene methylation correlations with patient variables. And in the third part we have analyzed whether the methylation status of five genes separately or in clusters are independent predictors for patient outcome.

## Methods

### Patient sample

The methylation profile of the *MGMTGATA6CD81DR4*, and *CASP8* genes in 76 patients with WHO grade IV glioblastomas was investigated. All glioblastomas were graded and classified according to the 2007 WHO classification criteria [5]. Tissue samples were collected from the Department of Neurosurgery, Hospital of Lithuanian University of Health Sciences Kaunas Clinics, from 2003 to 2009. The ethics committee of Lithuanian University of Health Sciences and the Lithuanian Bioethics Committee approved the collection and use of human brain tumor tissue samples. Written informed consent was obtained from all patients before the surgery. Survival time was collected for all cases and calculated from the time of surgery to death or censor.

### Methylation-specific PCR

Brain tumor tissue specimens after dissection were snap-frozen and stored in liquid nitrogen until analysis. Tumor DNA was extracted using ZR Genomic DNA™-Tissue MiniPrep (Zymo Research, USA) from 25-40 mg of frozen tissue according to the manufacturer’s instructions. The methylation status of *MGMTCD81GATA6DR4*, and *CASP8* gene promoters was determined by bisulfite treatment of DNA. An amount of 400 ng of DNA was used for bisulfite modification. DNA modification was done using the EZ DNA Methylation Kit (Zymo Research, USA), and all the procedures were carried out according to the manufacturer’s protocol. Bisulfite-treated DNA was eluted in 40 *μ*L distilled water and stored in –80 °C until PCR. Normal human blood lymphocyte DNA treated with bisulfite served as a negative control. As a positive control, standard Bisulfite Converted Universal Methylated Human DNA Standard (Zymo Research, USA) was used. Promoter methylation was detected by methylation-specific PCR (MSP). Each MSP reaction incorporated approximately 20 ng of bisulfite-treated DNA as a template. Specific for methylated and unmethylated DNA sequence primers are listed in Table 1 and were obtained from published data (for *MGMT*[6], *CD81*[7], *GATA6*[8], *DR4*[9], and *CASP8*[10]). PCR reaction was performed in a total volume of 20 *μ*L, using 10 *μ*L Maxima® Hot Start PCR Master Mix with Hot Start Taq DNA polymerase (Thermo Fisher Scientific, USA) and 10 *μ*M of each primer (Metabion International AG, Germany). MSP was performed for 38-40 cycles with start of 95 °C for 1 min, annealing for 1 min at temperature appropriate for an individual gene, and extension at 72 °C for 1 min. Amplification products were loaded on 2% agarose gels with ethidium bromide and after electrophoresis documented under UV. In a case of both, methylated and unmethylated signals appearance in a gel, methylation of the gene was considered.

**Table 1:** Primers for methylation-specific PCR

Gene	Forward primer 5´– 3´	Reverse primer 5´ – 3´	Tm
*MGMT-M*	TTTCGACGTTCGTAGGTTTTCGC	GCACTCTTCCGAAAACGAAACG	64
*MGMT-U*	TTTGTGTTTTGATGTTTGTAGGTTTTTGT	AACTCCACACTCTTCCAAAAACAAAACA	62
*CD81-M*	CGACGGCGGCGATTTTATCGC	GACCTACGAAACGCGAACCG	58
*CD81-U*	GTGATGGTGGTGATTTTATTGT	ACAACCTACAAAACACAAACCAA	58
*GATA6-M*	CGGGGTAGATTTCGGATTCGC	CAACCGAACCTCGAACGAACG	60
*GATA6-U*	GTGTGGGGTAGATTTTGGATTTGT	AAACAACCAAACCTCAAACAAACA	60
*DR4-M*	TTCGAATTTCGGGAGCGTAGC	GTAATTCAATCCTCCCCGCGA	60
*DR4-U*	GTAGTGATTTTGAATTTTGGGAGTGTAGT	CTCATAATTCAATCCCCACAA	60
*CASP8-M*	TAGGGGATTCGGAGATTGCGA	CGTATATCTACATTCGAAACGA	58
*CASP8-U*	TAGGGGATTTGGAGATTGTGA	CCATATATATCTACATTCAAAACAA	58

M = methylated, U = unmethylated, Tm = melting temperature.

### Statistical analysis

Statistical analysis was carried out with the software of IBM SPSS Statistics 19 (IBM SPSS Inc., Chicago, IL). Quantitative data presented as mean and standard deviation (SD). To show the reliability of the estimate, the confidence interval (CI) with 95% confidence level was presented. For testing the statistical hypothesis the significance level of 0.05 was selected. The Kaplan-Meier method was used to estimate survival functions. For comparison of survival between two groups, the log-rank and generalized Wilcoxon tests were used. The Cox proportional hazard regression model was applied to determine independent variables and prognosis relative hazard. For comparing means of two groups the independent samples *t* test was used. For testing statistical hypothesis about the independence of two variables, the chi-square test was used.

## Results

### Glioblastoma patient data

The mean age at diagnosis was 61.2 years (range: 34-88; SD: 12.3 years). The male-to-female ratio was 1:1.5. The mean age of male (n = 30) and female (n = 46) patients was 59.9 years (SD: 13.3 years) and 62 years (SD: 11.6 years), respectively.

The mean survival of patients with glioblastoma (n = 76) was 14.6 months (range: 0.26-52.60 months), while the median survival was 7.7 months (95% CI: 4.81-10.6 months). The survival rate at 12 and 24 months was 34.2% (95% CI: 23.7%-46.0%) and 22.4% (95% CI: 13.6%-33.4%), respectively.

As expected, it was observed glioblastoma patient survival association with age and adjuvant treatment (log-rank test, *P* = 0.001). The median survival of patients aged <60 years and ≥60 years was 15.6 and 4.5 months, respectively. Radiotherapy (RT) alone and RT plus concomitant chemotherapy (temozolomide) (RT + TMZ) were used for the postoperative treatment of glioblastomas in our study. RT alone was administered in 85.3% (n = 58) of cases and RT + TMZ was used in 14.7% (n = 10) of cases. Eight patients did not receive postoperative treatment due to their critical condition. The median survival for patients treated with RT + TMZ versus RT alone was 42.2 months (95% CI: 13.19-71.18 months) and 7.7 months (95% CI: 4.34-11.04 months).

### Frequency of gene methylation and co-methylation in glioblastoma

**Figure 1:**
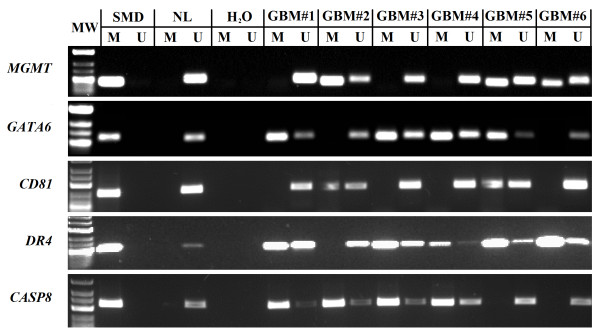
**Representative methylation specific PCR reaction for*MGMT, GATA6, CD81, DR4*and*CASP8*genes.** U represents amplification of unmethylated allele, and M represents methylated allele. Standard Bisulfite Converted Universal Methylated Human DNA (SMD) and normal human peripheral lymphocytes (NL) served as positive and negative methylation controls, respectively. MW – molecular weight. H_2_O – water control. GBM#1-6 glioblastoma patient tumor samples.

The overwhelming majority (97.3%) of tumors were methylated in at least one of five genes tested. Only 2.7% of glioblastomas had no detectable gene methylation, and 4.1% had concomitant methylation of all five genes tested. Methylation frequencies were 51.3% (n = 76) for *MGMT*, 68.4% (n = 76) for *GATA6*, 46.1% (n = 76) for *CD81*, 41.3% (n = 75) for *DR4*, and 56.0% (n = 75) for *CASP8*. Representative methylation-specific PCR for the mentioned genes is illustrated in Figure 1.

Despite the frequent co-methylation of two genes in the same tissue varying from about 15% to 37%, no significant associations in the frequencies of gene methylation were documented (chi-square test, *P* > 0.05).

### Associations between gene methylation and co-methylation, and patient data

Methylation of *MGMT* was associated with younger patient age at diagnosis (chi-square test, *P* = 0.039), while methylation of *CASP8* more often was observed in older patients (chi-square test, *P* = 0.002) (Table 2). When grouping patients according to survival time, it was shown that *MGMT* gene methylation was significantly more frequent event in patient group who survived longer than 36 months after diagnosis (chi-square test, *P* = 0.031 and *P* = 0.006, respectively), while *CASP8* and *DR4* gene methylation was more frequent event in patient group who survived up to 36 months (chi-square test, *P* = 0.035 and *P* = 0.061 respectively) (Table 2). *GATA6* and *CASP8* methylation in older patients were significantly associated with co-unmethylated *MGMT* (chi-square test, *P* = 0.021 and *P* = 0.003 respectively). Of the patients older than 60 years who lacked *MGMT* promoter methylation, 45.5% and 44.2% showed concomitant aberrant *GATA6* and *CASP8* methylation respectively. *GATA6* methylation in older patients was significantly associated with co-methylated *CASP8* and *DR4* (28% and 24.9%, chi-square test, *P* = 0.005 and *P* = 0.039 respectively), as well as *CASP8* methylation with co-methylated *DR4* (34.9%) (chi-square test, *P* = 0.017).

**Table 2:** Associations between demographic and clinical data of patients and methylation of five genes

Characteristic	n	*MGMT*			*GATA6*			*CD81*			*CASP8*			*DR4*		
		U (%)	M (%)	*P*	U (%)	M (%)	*P*	U (%)	M (%)	*P*	U (%)	M (%)	*P*	U (%)	M (%)	*P*
*Age*																
<60 years	32	14.5	27.6	**0.039**	15.8	26.3	0.454	22.4	19.7	1.000	28.0	14.7	**0.002**	26.7	14.7	0.477
≥60 years	44	34.2	23.7		15.8	42.1		31.6	26.3		16.0	41.3		32.0	26.7	
*Sex*																
Male	30	56.7	43.3	0.348	33.3	66.7	0.806	53.3	46.7	1.000	37.9	62.1	0.477	50.0	50.0	0.239
Female	46	43.5	56.5		30.4	69.6		54.3	45.7		47.8	52.2		64.4	34.6	
Survival																
<36 months	62	56.5	43.5	**0.006**	30.6	69.4	0.775	54.8	45.2	0.774	37.7	62.3	**0.035**	53.2	46.8	0.061
≥36 months	14	14.3	85.7		35.7	64.3		50.0	50.0		71.4	28.6		84.6	15.4	

n – number of patients, U = unmethylated, M = methylated.

### Associations between gene methylation and patient survival

**Figure 2:**
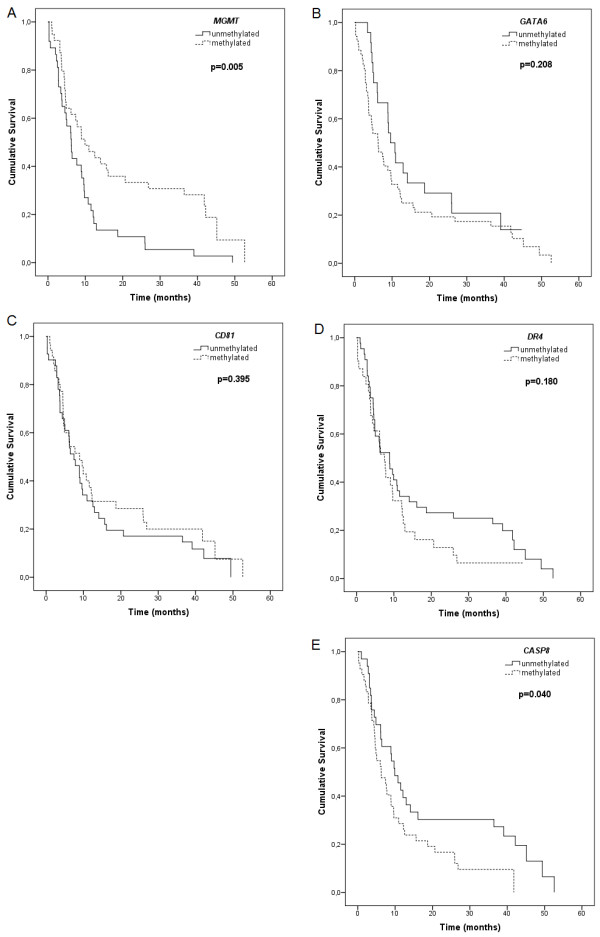
**Kaplan-Meier cumulative survival (months) in glioblastoma patients.** According to promoter methylation status of (A) *MGMT* gene (log-rank test, *χ*^2^=7.997, *df* = 1, *P* = 0.005), (B) *GATA6* gene (log-rank test, *χ*^2^ = 1.582, *df* = 1, *P* = 0.208), (C) *CD81* gene (log-rank test, *χ*^2^ = 0.719, *df* = 1, *P* = 0.395), (D) *DR4* gene (log-rank test, *χ*^2^ = 1.800, *df* = 1, *P* = 0.180), and (E) *CASP8* gene (log-rank test, *χ*^2^ = 4.214 *df* = 1, *P* = 0.040).

Figure 2 shows the associations between the methylation of five gene promoters and patient survival. The methylation of the *MGMT* promoter was associated with longer survival (log-rank test, *P* = 0.005) (Figure 2A). The median survival in the methylated and unmethylated *MGMT* groups was 9.9 months and 6.2 months, respectively (Table 3). The 12- and 24-month survival rates for methylated versus unmethylated *MGMT* cases were 46.2% (95% CI: 30.1%-62.8%) and 33.3% (95% CI: 19.1%-50.2%) versus 21.6% (95% CI: 9.8%-38.2%) and 10.8% (95% CI: 3.0%-25.4%), respectively.

**Table 3:** Kaplan-Meier survival function analysis in glioblastoma patients (log-rank test)

Variable		n	Events	Survival (months)		
				Median	95% CI	*P*
*MGMT* promoter methylation	U	37	37	6.2	4.6 to 7.9	**0.005**
	M	39	32	9.9	4.2 to 15.7	
*GATA6* promoter methylation	U	24	20	9.6	7.2 to 11.9	0.208
	M	52	49	6.2	2.9 to 9.5	
*CD81* promoter methylation	U	41	38	7.5	4.6 to 10.3	0.395
	M	35	31	8.9	4.6 to 13.3	
*DR4* promoter methylation	U	44	40	8.9	4.9 to 12.8	0.174
	M	31	29	7.5	5.6 to 9.3	
*CASP8* promoter methylation	U	33	29	9.9	6.9 to 12.9	**0.040**
	M	42	39	6.2	3.3 to 9.2	

n – total number of patients; M – methylated; U – unmethylated.

No significant associations between the methylation of *GATA6*, *CD81*, and *DR4* and patient survival were identified (Figures 2B–D). However, analysis of patient survival at early postoperative period showed a significantly shorter survival among patients with the methylated *GATA6* promoter (Wilcoxon test, *P* = 0.045).

Contrary to the methylation profile of *MGMT*, the methylation of the *CASP8* promoter was associated with shorter survival (log-rank test, *P* = 0.040) (Figure 2E). The median survival among patients with the unmethylated *CASP8* promoter was 9.9 months (95% CI: 6.9-12.9 month) as compared with 6.2 months (95% CI: 3.3-9.2 months) among their counterparts with the methylated *CASP8* promoter.

### Associations between gene co-methylation and patient survival

**Figure 3:**
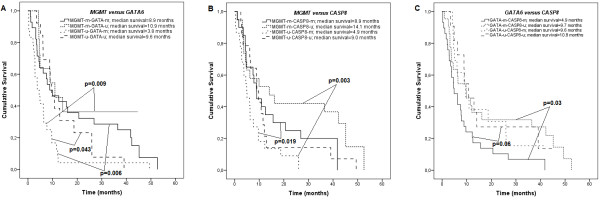
**Kaplan-Meier cumulative survival (months) in glioblastoma patients plotted according to concomitant gene methylation patterns.** (**A**) *MGMT* and *GATA6* gene methylation status (log-rank test, *χ*^2^ = 13.058, *df* = 3, *P* = 0.005), (**B**) *MGMT* and *CASP8* gene methylation status (log-rank test, *χ*^2^ = 12.314, *df* = 3, *P* = 0.006), (**C**) *GATA6* and *CASP8* gene methylation status (log-rank test, *χ*^2^ = 7.712, *df* = 3, *P* = 0.052).

Concomitant gene methylation effect on patient survival was done on genes significantly associated with survival in the univariate analysis. Kaplan-Meier analysis showed that not methylated *MGMT* in a case of methylated *GATA6* and *CASP8* were significantly associated with worse outcome (median survival – 3.8 months and 4.9 months, respectively) as compared to that of the patients whose tumors had methylated *MGMT* with unmethylated *GATA6* and *CASP8* (median survival – 10.9 and 14.1 months respectively, log-rank test, *P* = 0.009 and *P* = 0.003 respectively), as well as tumors with no methylation of both genes (*MGMT* and *GATA6*) (median survival – 9.6 months, log-rank test, *P* = 0.043), and tumors with double methylation (*MGMT* and *CASP8, MGMT* and *GATA6*, median survival – 8.9 and 8.9 months, log-rank test, *P* = 0.019 and *P* = 0.006 respectively) (Figure 3A-B). Concomitant methylation status analysis of *GATA6* and *CASP8* showed the lowest median survival in a group of patients with both methylated genes in a tumor (median survival – 4.9 months), and the difference was significant as compared with patients whose tumors had methylated *GATA6* and unmethylated *CASP8* (median survival – 9.7 months; log-rank test, *P* = 0.03) (Figure 3C).

Univariate Cox analysis identified patient age, treatment modality, and methylation profile of the *MGMT* and *CASP8* promoters as independent predictive factors significantly associated with patient survival. Patients older than 60 years were more likely to survive shorter than their younger counterparts (hazards ratio [HR]: 1.06; 95% CI: 1.04-1.09; *P* = 0.001). Moreover, patients who underwent treatment with RT + TMZ had a 76% reduced risk of death (HR: 0.24, 95% CI: 0.09-0.60; *P* = 0.003) as compared with patients treated with RT alone.

The methylation status of *MGMT*, *CASP8*, and *GATA6* was of prognostic value for survival in a multivariate Cox model, when patient age and treatment were excluded (Table 4). The methylated *MGMT* promoter was associated with longer survival (HR: 0.45, 95% CI: 0.27-0.76; *P* = 0.002). Contrary, the methylated *GATA6* and *CASP8* promoters were found to be associated with shorter survival (HR: 1.77 95% CI: 1.02-3.05; *P* = 0.041 and HR: 1.84 95% CI: 1.05-3.22; *P* = 0.033, respectively).

**Table 4:** Multivariate Cox regression survival analysis for glioblastoma patients

Variable	Multivariate Cox regression		
	HR	95% CI	*P*
*MGMT*	0.45	0.27-0.76	**0.002**
*GATA6*	1.77	1.02-3.05	**0.041**
*CD81*	0.69	0.41-1.15	0.168
*CASP8*	1.84	1.05-3.22	**0.033**
*DR4*	1.09	0.65-1.83	0.755

HR – hazard ratio, CI – confidence interval.

When the patient sample was divided into two groups by age (<60 years and ≥60 years), multivariate Cox regression analysis showed that *MGMT* (HR: 0.15, 95% CI: 0.05-0.42; *P* = 0.001) and *GATA6* (HR: 2.78, 95% CI: 1.01-7.65; *P* = 0.047) were independent predictors for patient survival in younger patients’ group, while there were no significant associations observed in older patients’ group when adjusted for therapy (data not shown).

## Discussion

Our study demonstrated that epigenetic alterations affecting multiple genes, which are important in several cell functions, such as DNA repair, cell migration and apoptosis, are a frequent event in glioblastoma. At least one of the five genes tested was methylated even in 97.3% of patients with glioblastoma, while only 2.7% of patients had no detectable methylation. Analysis identified 3 genes epigenetically deregulated in more than 50% of glioblastomas: *MGMT*, *GATA6*, and *CASP8*. To our knowledge, only *MGMT* has been previously widely described in glioblastomas, while the data on the epigenetic regulation of *GATA6*, *CD81*, *DR4*, and *CASP8* in patient glioblastoma are scarce.

This study showed that the most frequently methylated gene was *GATA6*, a transcription factor, with a methylation frequency of 68.4%. *GATA6* is one of 6 members of the mammalian *GATA* family of transcription factors that regulates cell proliferation and differentiation and inhibits apoptosis [11]. Recently, it has been suggested as a tumor suppressor gene in tumors of the CNS with gene expression loss of 90% and promoter methylation of 30%-48% in glioblastomas [3,12-14]. It has been reported that loss of GATA6 results in enhanced astrocyte proliferation and transformation [14]. A decrease in *GATA6* expression was observed in colon and ovarian carcinomas as well [15,16].

The *CD81* gene showed promoter methylation in 46.1% of tested glioblastomas in the current study. This gene is a member of the membrane-embedded tetraspanin superfamily, which was found to be silenced by methylation in multiple myeloma cell lines [7]. *CD81* participates in several functions in a cell like adhesion and signal transduction [17]. Recent glioblastoma methylome studies have shown *CD81* methylation rate of 54% [2,3].

Analysis of the methylation status of two proapoptotic genes, *CASP8* and *DR4*, in our study revealed that these genes were methylated in 56% and 41% of glioblastomas, respectively. The inactivation of these genes by promoter methylation has been previously reported in osteosarcomas [18], melanomas [19], medulloblastomas [20], gastric carcinomas [9], and glioblastomas [3,13,21,22]. The frequency of *DR4* methylation in glioblastomas varied from 25% to 70% in different studies [3,21,22]. In our series as compared with the above studies, a lower frequency of *DR4* methylation was observed, and we hypothesize that the difference could be attributed to the heterogeneity of glioblastomas. In contrast to the findings of Elias et al. [21] and Hervouet et al. [13] who showed 10%-30% methylation of *CASP8* in glioblastomas, in this panel more than half of tumors were methylated (56%). The tendency of association between methylation of *CASP8* and *DR4* in glioblastomas was noted: co-methylation was observed in 28.4% (21/74) of tumors. A study carried out by Elias et al. [21] reported a smaller percentage of *CASP8* and *DR4* co-methylation in glioblastoma specimens (10%). It is known that *DR4* and *CASP8* are factors affecting the nonmitochondrial apoptotic pathway, and the loss of *DR4* expression (which was shown to be mediated by promoter methylation) attenuates apoptosis and is associated with the resistance of glioma cells to proapoptotic ligand therapy (known as TRAIL resistance) [21]. The importance of *CASP8* in TRAIL resistance in gliomas has been reported as well [23], while the data of other studies have strongly suggested TRAIL sensitivity to be *CASP8*-independent and *DR4*-specific [21].

Epigenetic silencing of the *MGMT* gene, encoding a DNA repair enzyme, has been recently found to be of predictive value in glioblastoma. In agreement with numerous studies where the methylation of *MGMT* was detected in approximately 40%-48% of primary glioblastomas [1,2,24], our study showed a methylation frequency of 51%. However, contrary to the study by Cecener et al. [12], this study found no significant association between the methylation of *MGMT* and *GATA6* (*P* > 0.05) despite rather high comethylation of these genes (37%).

Correlations with patient parameters showed *MGMT* and *CASP8* gene methylation associations with patient age. Glioblastoma patients with methylated *MGMT* were significantly younger than those whose tumor lacked methylation (*P* < 0.05) and an opposite was observed for *CASP8* (*P* < 0.01). Contrary, glioblastoma study of Cecener et al. [12] showed *MGMT* methylation association with older patient age (≥ 50 years), while in the study of Weller et al. [24]*MGMT* methylation status was not associated with clinical parameters (age, extent of resection, Karnofsky performance score or treatment).

Several associations between survival and patients’ characteristics were observed. Survival was strongly correlated with patients’ age and treatment modality. In agreement with previous studies [24,25], it was observed that glioblastoma patients younger than 60 years and treated with RT + TMZ survived longer as compared with older and only RT-treated patients. Weller et al. [24] revealed age as a major therapy-independent factor in patient survival.

Cox regression analysis confirmed the methylation status of *MGMTGATA6*, and *CASP8*, but not *CD81* and *DR4*, to be an independent factor for patient survival. Kaplan-Meier and Cox analysis showed the methylation status of *MGMT* to be significantly related to patient outcome. In agreement with previous studies [1,3,26] reporting the associations between *MGMT* methylation status and patient survival, our study showed that patients with methylated *MGMT* were more likely to survive longer and were at lower risk of death than those with the unmethylated gene. After dividing the patient sample into the groups by age (<60 years vs. ≥60 years), methylated *MGMT* remained a significant independent predictor of survival among patients aged less than 60 years. Furthermore, we confirmed significant association between the methylation profile of *MGMT* and treatment modality: in the group of patients treated with RT + TMZ, patients with methylated *MGMT* survived significantly longer than those with unmethylated gene promoter (median survival, 41.4 vs. 15.1 months) (data not shown).

In line with findings of the study by Martinez et al. [22], associations between the methylation of *CASP8* and survival in our study showed gene methylation being an important factor for worse outcome of patients. Patients with unmethylated *CASP8* as a favorable genotype had prolonged survival as compared with patients having tumors with methylated *CASP8*. Although *CASP8* may play an essential role in apoptosis induced by chemotherapeutic agents and radiation therapy [27,28], our study showed only a trend toward significant associations between the methylation status of *CASP8* and survival of the patients treated with RT, and no associations in the group of the patients treated with RT + TMZ (data not shown).

Survival among patients with the methylated vs. unmethylated *GATA6* gene differed significantly at early postoperative period (3-month survival, 100% vs. 75%). Cox regression analysis demonstrated *CASP8* and *GATA6* as independent predictors for patient survival. Furthermore, *GATA6* became independent predictor for survival in younger patient group. Contrary to our results, recently it have been reported no relationship of *GATA6*[12] and *CASP8*[13] methylation status with survival in patients with glioblastoma. In agreement with the methylome study by Martinez et al. [3], where the significance of *GATA6* methylation in patient survival has been shown for the first time, our results suggest that the methylation of *GATA6* is a frequent event and highly important for survival of patients with glioblastoma.

## Conclusions

The current study reveals new and important information on *MGMT*, *GATA6* and *CASP8* promoter methylation in glioblastoma. *GATA6* methylation occurred at a highest rate. Patient age, treatment regimen, *MGMT*, *GATA6* and *CASP8* methylation status were significantly associated with patient survival. *MGMT* and *GATA6* were independent predictors for patient survival in younger patients’ group when adjusted for therapy. To further evaluate validation of the methylation profile of these genes is necessary for understanding their role in gliomagenesis and potential as GBM markers.

## Competing interests

The authors are not aware of any biases that might be perceived as affecting the objectivity of this article.

## Authors’ contributions

DS, PV, and VA generated an idea. DS did a part of *DR4*, *MGMT* and *GATA6* MSP analysis, statistical analysis and wrote a paper. PV did *CD81* and *CASP8* methylation analysis. VS did all statistical analysis. AT, KS, PVD generated an idea, gathered postoperative glioblastoma tissue and patient data.

## Pre-publication history

The pre-publication history for this paper can be accessed here:

http://www.biomedcentral.com/1471-2407/12/218/prepub
